# From empirical screening to lipid trolling: the evolution of extrahelical allosteric modulators of A_1_ and A_3_ adenosine receptors

**DOI:** 10.1007/s00044-026-03534-5

**Published:** 2026-03-16

**Authors:** Matteo Pavan, Siva Hariprasad Kurma, Paola Oliva, David P. Broughton, Zhan-Guo Gao, John A. Auchampach, Kenneth A. Jacobson

**Affiliations:** 1 Molecular Recognition Section, Laboratory of Bioorganic Chemistry, National Institute of Diabetes and Digestive and Kidney Diseases, National Institutes of Health, Bethesda, MD USA; 2 Department of Pharmacology & Toxicology and the Cardiovascular Center, Medical College of Wisconsin, Milwaukee, WI USA

**Keywords:** GPCR, Lipid trolling, Allosteric, Ago-PAM, Molecular dynamics

## Abstract

An early GPCR positive allosteric modulator (PAM) was the 2-amino-3-benzoylthiophene derivative PD81,723, which enhances G_i_-coupled A_1_ adenosine receptor (AR) agonist effects. Cryo-EM studies located its extrahelical binding site on TMs 5 and 6, and extensive empirical SAR is consistent with the experimental A_1_AR structure. At the anti-inflammatory A_3_AR, a series of 1*H*-imidazo[4,5-*c*]quinolin-4-amine PAMs, originally derived from an A_1_AR antagonist scaffold, was recently found, using mutagenesis and molecular modeling, to bind at an extrahelical, lipid-exposed site on the receptor. The *N*4 and C2 positions on the scaffold were modified with substituted phenyl and cycloalkyl/branched alkyl groups, respectively, as in the prototypical PAM LUF6000. Before the structural binding hypothesis, extensive synthetic optimization failed to introduce hydrophilic groups needed for aqueous compatibility. Eventually, secondary anchoring of these A_3_AR PAMs within the phospholipid bilayer (lipid trolling) succeeded in improving the pharmacological profile, based on the attraction of appended terminal cationic chains on the PAM with anionic phospholipid head groups. Molecular dynamics (MD) simulation has predicted a rearrangement of anionic lipids around H8 when long-amino chain PAMs bind, and an upward movement of H8 that is reported to be associated with Class A GPCR activation. *N*1 alkylation with benzyl groups removed residual A_3_AR antagonism and produced substantial enhancement of agonist potency as well as E_max_. Both A_1_AR and A_3_AR PAMs can activate partially in the absence of orthosteric agonist. Thus, at both receptors, potent PAMs have been discovered and optimized for selective enhancement of endogenous adenosine and potentially for treatment of conditions such as chronic pain. Collectively, extrahelical, lipid-exposed pockets are druggable allosteric sites in Class A GPCRs and amenable to deliberate engagement of the membrane environment to optimize receptor modulation. The evolution from empirical screening to structure-guided and membrane-aware design provides a generalizable framework for targeting signaling across the GPCR superfamily beyond ARs.

**Figure:**
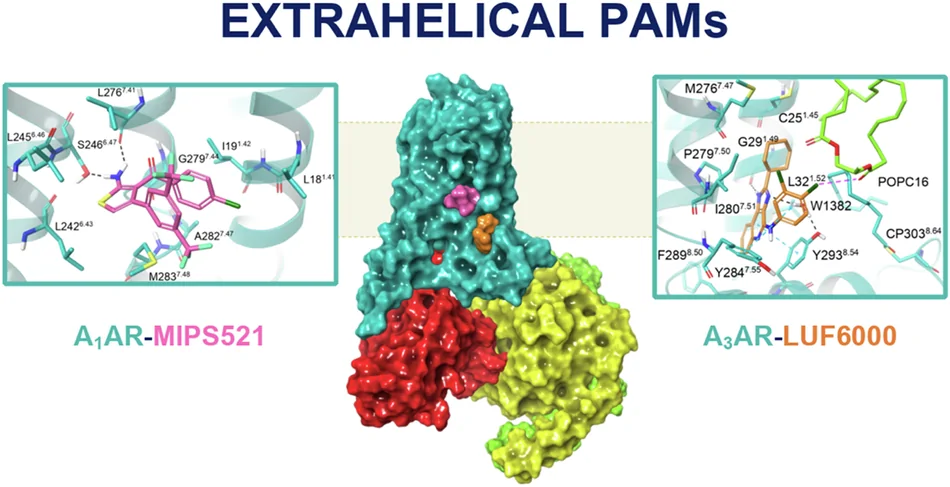

## Introduction

Adenosine plays a role in regulating numerous physiological processes, including cardiovascular function, immune response, and neurotransmission [1]. The A_1_ and A_3_ subtypes of adenosine receptors (ARs) are G_i_ protein-coupled and complement the G_s_-coupled A_2A_ and A_2B_ ARs. Extensive medicinal chemical studies of ligands for these receptors have been conducted since the 1980s, resulting in numerous pharmacological tool compounds and clinical candidate molecules [1]. Most of these studies have revealed the structure-activity relationship (SAR) of orthosteric agonists and antagonists. However, some of the reported ligands were found to be allosteric modulators. In particular, two major families of A_1_AR and A_3_AR positive allosteric modulators (PAMs) have been extensively explored [2, 3].

**Fig. 1:**
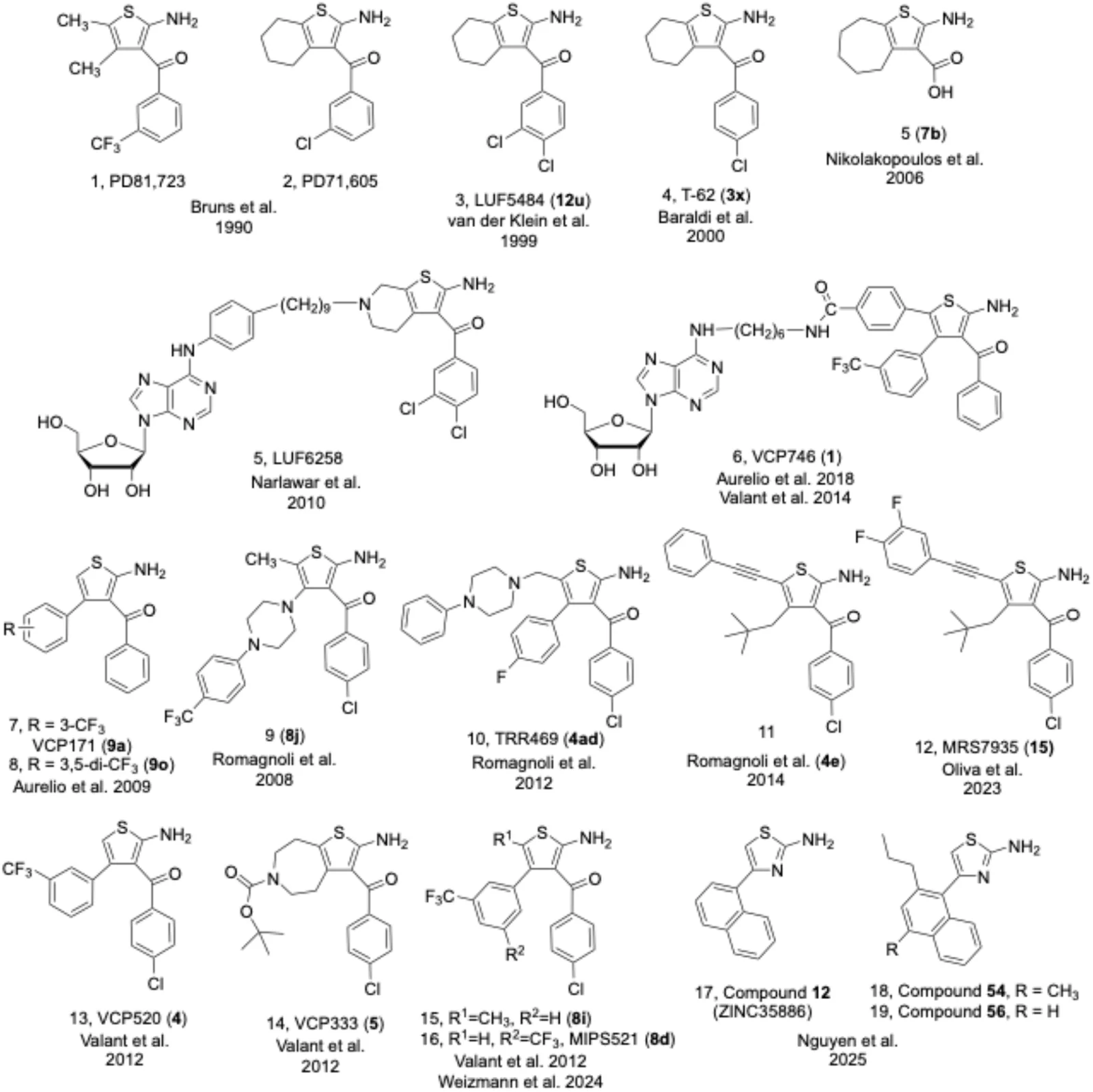
A_1_AR PAMs derived from the prototypical PAM PD81,723 1. Bold numbers in parentheses refer to the compound number in the cited publication [2, 20–22, 27, 29, 31, 36, 44, 45, 73, 79, 101, 102]

**Fig. 2:**
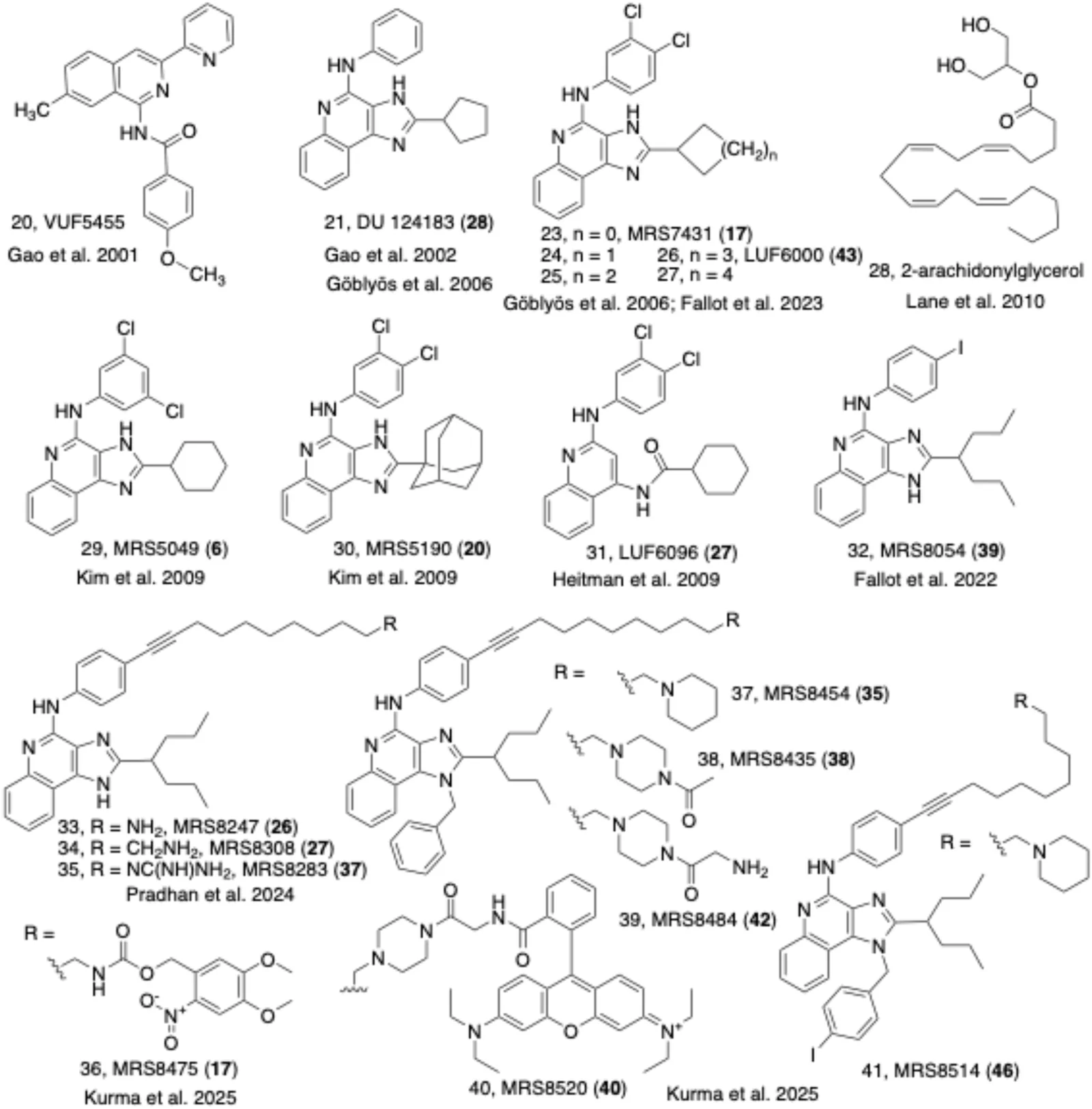
A_3_AR PAMs of three different chemotypes and one NAM **28**. Most are derived from the originally identified 1*H*-imidazo[4,5-*c*]quinolin-4-amines PAM, e.g. DU124183 **21** and prototypical PAM LUF6000 **26**. Bold numbers in parentheses refer to the compound number in the cited publication [8–10, 12, 58, 60, 61, 103]

Allosteric modulation of GPCRs, by interacting at a site different than that of the native agonist, is an attractive alternative to orthosteric agonists and antagonists [4–6]. While orthosteric agonists would tend to activate the receptor wherever it occurs, positive allosteric modulators (PAMs) preferentially enhance receptor activity only in the presence of elevated endogenous agonists, such as adenosine, under specific physiological or pathological conditions. One of the earliest examples of GPCR PAMs to be discovered was the 2-amino-3-benzoylthiophene series, which enhances A_1_AR agonists. The prototypical A_1_AR PAM PD81,723 **1** (fig. 1) was reported in 1990 by R. Fred Bruns and colleagues at Parke-Davis (now Pfizer) [2]. PD81,723 **1** is a 2-amino-3-benzoylthiophene derivative that increased binding of the A_1_AR radioligand [^3^H]CHA (*N*^6^-cyclohexyladenosine) to the A_1_AR in rat brain cortical membranes, with a maximal increase of approximately 25% over baseline. Two heterocyclic A_3_AR PAM chemotypes were discovered in the early 2000s (fig. 2) by our lab in collaboration with Ad IJzerman of Leiden University, as described in Gao et al. [3, 7, 8]. These chemotypes include 1*H*-imidazo[4,5-*c*]quinolin-4-amines [7–9], notably DU124183 **21** and LUF6000 **26**, and 3-(2-pyridinyl)isoquinolines [8], exemplified by VUF5455 **20**. Both classes were shown to enhance A_3_AR orthosteric agonist radioligand binding and agonist-induced guanine nucleotide [^35^S]GTPγS binding, i.e., a functional assay of receptor activation, and to reduce the off-rate of orthosteric agonist [^125^I]I-AB-MECA (*N*^6^-(4-amino-3-iodobenzyl)adenosine-5′-*N*-methyluronamide). A third chemotype, 2,4-disubstituted quinolines, e.g., LUF6096 **31**, representing a ring-opened amide version of the imidazoquinolinamine **26**, was later reported by Heitman et al. [10]. Notably, this PAM showed anti-ischemic cardioprotective effects in vivo in dogs [11]. However, LUF6096 was found to be rapidly cleared from plasma in this study. Curiously, a lipid derivative, arachidonyl glycerol **28**, was also identified as a weak A_3_AR NAM [12].

Although there are now experimental structures of both the A_1_AR [13] and A_3_AR [14], these classes of PAMs were identified initially by empirical screening, using radioligand binding, rather than by structure-based approaches. It is to be noted that both classes, as optimized, are specific in binding to their target receptor, with no off-target interaction at other ARs. However, the location of both major PAM classes has now been established, either through cryo-electron microscopic (cryo-EM) structures (A_1_AR) or through detailed molecular modeling, including molecular dynamics (MD) simulations supported by mutagenesis and SAR (A_3_AR). Unexpectedly, both the 2-amino-3-benzoylthiophenes and the 1*H*-imidazo[4,5-*c*]quinolin-4-amines were found to bind at extrahelical and lipid-exposed sites on the A_1_AR and A_3_AR, respectively. The structural insights into the binding site environment (see below) have informed recent SAR studies of both A_1_ and A_3_ PAMs. In general, lipophilic ligands that bind to the protein-lipid interface are gaining importance as a means of increasing specificity for a particular target and overcoming resistance mechanisms [15, 16].

**Fig. 3:**
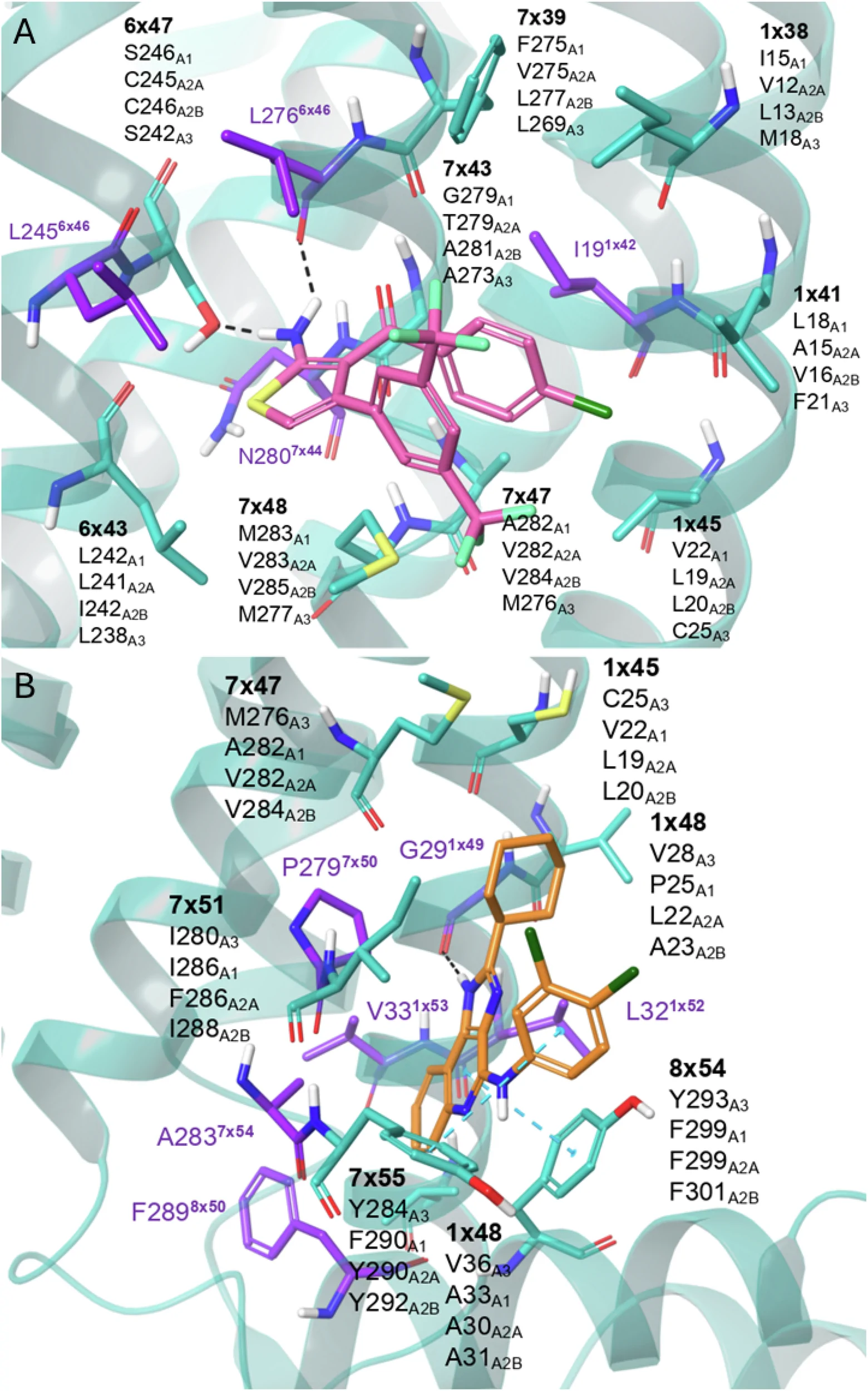
Comparison of allosteric binding site residues of the A_1_AR (A, binding site of MIPS521 **16** in the cryo-EM structure, PDB ID: 7LD3 [17]) and the A_3_AR (B, modeled binding site of LUF6000 **26** [60] across different AR subtypes. Conserved residues are highlighted in purple. For each residue, a generic structure-based numbering is reported [104], that accounts for helical bulges and constrictions

Many clinical trials evaluating orthosteric A_1_AR agonists for conditions including pain and metabolic disorders have been conducted; however, nearly all have been discontinued to date [1] most often due to unacceptable side effect profiles. Clinical trials of A_3_AR agonists for autoimmune/inflammatory conditions (specifically psoriasis, metabolic dysfunction-associated steatohepatitis (MASH), and canine osteoarthritis), cancer, and brain ischemia are ongoing [17, 18], although earlier trials of A_3_AR agonists for different conditions (rheumatoid arthritis, dry eye syndrome, glaucoma) were discontinued. The advantage of applying PAMs for receptor activation over orthosteric agonists has been described [4–6]. When the endogenous agonist, i.e., extracellular adenosine, is elevated under stressed and pathological conditions, a PAM amplifies the adenosine effect. When adenosine concentrations are at basal, low levels, the effect of a PAM would be absent or subthreshold. This could contribute to greater spatiotemporal selectivity of action. Because the A_1_ and A_3_ ARs are widely distributed throughout the body, side effects remain likely even with highly selective agonists. The levels of endogenous adenosine rise dramatically in cases of ischemia, chronic pain, tissue inflammation, and other stressed states, suggesting that PAMs could be clinically efficacious. Both A_1_ and A_3_ AR agonists have anti-ischemic effects and attenuate chronic pain. Moreover, the two PAM classes discussed here exhibit high AR subtype selectivity, due to their interaction with distinct allosteric binding sites that are not conserved across AR subtypes (fig. 3, details discussed below). Thus, in principle, a PAM with more selective action than a direct-acting orthosteric agonist would be advantageous.

## Discovery and optimization of A_1_AR PAMs

The prototypical A_1_AR PAM PD81,723 (2-amino-4,5-dimethylthiophen-3-yl)(3-(trifluoromethyl)phenyl)methanone) **1** was found to increase agonist radioligand binding, but also inhibit antagonist radioligand binding [2], suggesting that PD81,723 binding to its allosteric site stabilizes the agonist-bound, active conformation of the A_1_AR. Furthermore, **1** and its analogues exhibit intrinsic agonist activity, a property referred to as ago-PAM activity [13, 19]. The SAR of PAMs related to **1** based on key analogues is summarized in fig. 1, with the activity of selected compounds displayed in Table 1 [20–22]. A variety of pharmacological approaches have been employed to probe allosteric effects, including equilibrium binding, kinetic binding, and function-based approaches, as well as strategies to isolate specific signaling pathways. In Table 1, data for four compounds demonstrated robust enhancement of A_1_AR agonist-induced ERK1/2 phosphorylation [20]. The data for four additional compounds demonstrate increased agonist radioligand binding [21, 22].

**Table 1:** Allosteric effect of representative A_1_AR PAMs on (upper) agonist-stimulation and on ERK1/2 phosphorylation and (lower) agonist and antagonist radioligand binding.

Compound	% maximal ERK activity, control^a^		% maximal activity, 3 µM^a^			% maximal activity, 10 µM^a^	
**7**	50		83 ± 3			59 ± 1	
**8**	50		83 ± 7			93 ± 8	
**13**	50		64 ± 8			73 ± 8	
**15**	50		94 ± 15			94 ± 4	
**16**	50		69 ± 9			74 ± 7	

Compound	[^3^H]CCPA B_max_^b^	[^3^H]CCPA+PAM, B_max_^c^		[^3^H]DPCPX B_max_^d^	B_max_ shift^e^		% of increase^f^
**1** PD81,723	101 ± 9	121 ± 11		873 ± 81	1.20		2.6
**10** TRR469	107 ± 11	706 ± 62		888 ± 82	6.60		76.7
**11**	102 ± 10	481 ± 37		877 ± 76	4.72		48.9
**12**	108 ± 10	667 ± 58		881 ± 70	6.18		72.4

^a^Effect on A_1_R-mediated stimulation of ERK1/2 phosphorylation in intact CHO cells in the presence of a EC_50_ concentration of R-*N*^6^-phenylisopropyladenosine (R-PIA, measured daily, typically 100-fold greater potency than the cAMP inhibition), Valant et al. [20].

^b^B_max_ values (fmol/mg protein) in agonist [^3^H]CCPA (2-chloro-*N*^6^-cyclopentyladenosine) saturation binding in the absence of PAM, assuming a K_D_ value of 1.17 ± 0.11 nM. N = 3, Oliva et al. [21].

^c^B_max_ values obtained from [^3^H]CCPA saturation binding experiments in the presence of a PAM (10 µM). N = 3.

^d^B_max_ values (fmol/mg protein) obtained from saturation binding experiments in the absence of a PAM by using antagonist [^3^H]DPCPX as radioligand. N = 3.

^e^Represents the ratio of B_max_ ([^3^H]CCPA + PAM) / B_max_ ([^3^H]CCPA).

^f^Percentage of increase calculated according to the following formula:

[B_max_ ([^3^H]CCPA + PAM) - B_max_ ([^3^H]CCPA) / B_max_ ([^3^H]DPCPX) - B_max_ ([^3^H]CCPA)] × 100.

Refer to Fig. 1 for structures.

The concept of GPCR biased agonism has been explored for about two decades [23]. The recent advances hold the potential to revolutionize GPCR-targeted drug discovery and promise more precise and effective treatments [24]. However, few, if any orthosteric A_1_AR agonists showed biased agonism [25]. In principle, biased agonism could more easily result from allosteric modulators, including allosteric agonists [26].

The SAR of 2-aminothiophenes as A_1_AR PAMs has been extensively investigated by multiple research groups [20–22, 26–42] and has been comprehensively reviewed by Nguyen et al. [43]. Both the 2-amino substituent and the thiophene core are critical for activity. However, various aza substitutions of the thiophene ring are tolerated, yielding thiazole analogs that can exhibit either increased [40] or reduced [41] potency relative to the parent thiophenes. Substitution of selenium for sulfur resulted in improved potency; however, the resulting selenophenes were unstable in mildly acidic conditions due to ring opening [39]. Until recently, the presence of a 3-benzoyl or other acyl group was also considered a defining feature of A_1_AR PAMs. The 3-benzoyl group accommodates various hydrophobic substituents at the 3,4 positions, and replacement of the benzoyl moiety at the 3-position of the thiophene with hydrophobic heteroaryl groups was well tolerated [38]. In addition, 3-carboxythiophene 3-trifluoromethylbenzyl esters and 3-(1-naphthoyl) derivatives were found to be active [42]. The 4,5-dimethyl groups are often cyclized, as in compounds **3**–**5**, with six-membered rings conferring greater potency than five-membered rings [37]. The 4-methyl group can also be replaced with branched alkyl, heterocycloalkyl, or hydrophobic-substituted phenyl groups, while the 5-methyl group may be deleted (**7,** **8,** **13,** **16**) or substituted with aryl-alkynyl moieties (**3**–**5**) or, as in **10**, with the piperazine-linked hydrophobic moieties. Notably, when switching to the thiazole scaffold in **17**–**19** [36, 43], the requirement for a 3-acyl group was eliminated; instead, simple phenyl or naphthal-1-yl groups were sufficient to provide high PAM activity.

Nucleosides conjugated to A_1_AR PAMs were designed to bind simultaneously to the allosteric and orthosteric binding sites, with the goal of achieving high PAM activity. However, recent structural insights have revealed that these conjugates (**5** and **6**) [44, 45] are unlikely to be able to extend between the two binding sites. Also, effective engagement of the A_1_AR allosteric site appears to require hydrophobic substituents to permit membrane embedding of the PAM molecule.

Partial A_1_AR agonists might display greater event- and site-specificity than full orthosteric agonists and thus have lower side effect liability, due to the higher efficacy associated with intended target tissues having higher receptor expression levels [46]. It was speculated that partially efficacious PAMs or ago-PAMs, such as **4**, might also have this advantage [47, 48]. T62 **4** reduced neuropathic pain hypersensitivity in vivo [49–51] and entered clinical trials (clinicaltrials.gov, NCT00809679, NCT00506610), which unfortunately failed to show efficacy against postherpetic neuralgia [52]. Other A_1_ PAMs were protective in other pain and ischemia reperfusion models [53]. A more potent A_1_AR PAM, TRR469 **10**, which protected against neuropathic pain in vivo [32], was under consideration as a treatment of anxiety-related disorders [28]. Like **1**, both **4** and **10** activated the A_1_AR alone as partial agonists in the absence of added orthosteric agonist, as shown using cAMP inhibition and [^35^S]GTPγS binding [2, 27, 32, 54].

In Nguyen et al. [36], the modulation of NECA potency and affinity by 2-amino-thiazole derivative **17** (hit from virtual screening of the ZINC database, Compound 12) was further investigated. The computationally derived PAMs **18** and **19** (Compounds 54 and 56) enhanced neuronal A_1_AR activity in vivo without unwanted cardiac effects, a common hindrance in AR agonist therapeutics.

## Discovery and optimization of A_3_AR PAMs

The first A_3_AR PAM to be reported [3] was the series of 1*H*-imidazo[4,5-*c*]quinolin-4-amines represented by LUF6000 **26**, which is under consideration for treatment of erectile dysfunction [9, 55]. LUF6000 was shown to enhance the genetic and functional modulation by an A_3_AR agonist in HL-60 cells that express a native A_3_AR [56]. The original hit compound, DU124183 (fig. 2, **21**), was part of a library of A_1_AR antagonists [57], and some AR antagonism remained in the series along with PAM activity. For example, **21** binds to human A_1_ and A_2A_ARs with K_i_ ~ 3 µM [9]. Subsequent medicinal chemistry campaigns improved the extent of A_3_AR agonist enhancement, most commonly assessed using the selective agonist Cl-IB-MECA (2-chloro-*N*^6^-(3-iodobenzyl)adenosine-5′-*N*-methyluronamide, Table 2). The prototypical A_3_AR PAM LUF6000 **26**, reported in 2006 [9], lacks A_1_, A_2A_ and A_2B_AR affinity. However, early derivatives of this chemical class were found to enhance agonist efficacy (E_max_) but not agonist potency (EC_50_). Moreover, some analogs within the series, such as 2-cyclopropyl derivative MRS7431 **23**, only produced inhibitory effects on A_3_AR signaling, acting as competitive antagonists rather than PAMs [58, 59]. Efforts to introduce polar substituents to improve aqueous solubility uniformly resulted in loss of PAM activity. The various rounds of SAR studies further revealed that chemical modification of the 1*H*-Imidazo[4,5-*c*]quinolin-4-amine scaffold were restricted to the C2 (cyclohexyl in LUF6000) position and the *N*4 amino substituent (*N*-3,4-dichlorophenyl in LUF6000), and that no hydrophilic groups were tolerated at either site.

**Table 2:** Allosteric Effect of 1*H*-Imidazo[4,5-*c*]quinolin-4-amines (1 µM) on Cl-IB-MECA-Induced A_3_AR Activation


Compound (R^2^)	R^2^	R^1^	E_max_^a^ (%) at 1.0 µM	pEC_50_^a^	EC_50_ ratio^b^
**26** (LUF6000)	3,4-Cl_2_		**217** ± 8^*^	7.42 ± 0.11	0.44
**32**	4-I		**223** ± 10^*^	7.34 ± 0.12	1.53
**33**	4-C≡C-(CH_2_)_8_NH_2_		**218** ± 5^*^	**8.11** ± 0.08^*^	5.11
**34**	4-C≡C-(CH_2_)_9_NH_2_		**238** ± 8^*^	8.23 ± 0.14	4.29
**35**	4-C≡C-(CH_2_)_8_NC(NH)NH_2_		**214** ± 17^*^	8.29 ± 0.29	9.44
**36**	4-C≡C-(CH_2_)_9_NH-NVOC		100 ± 4	7.37 ± 0.13	0.72
**37** (R^3^ = Bn)	4-C≡C-(CH_2_)_9_-piperidine		**273** ± 8^*^	**8.61** ± 0.13^*^	14.4
**38** (R^3^ = Bn)	4-C≡C-(CH_2_)_9_-piperazine-CO-CH_3_		**292** ± 16^*^	8.13 ± 0.17	2.75
**39** (R^3^ = Bn)	4-C≡C-(CH_2_)_9_-piperazine-CO-CH_2_NH_2_		**249** ± 11^*^	**8.34** ± 0.18^*^	10.7
**40** (R^3^ = Bn)	4-C≡C-(CH_2_)_9_-piperazine-CO-CH_2_NH-rhodamine B		**155** ± 6^*^	7.69 ± 0.11	1.95
**41** (R^3^ = 4-I-Bn)	4-C≡C-(CH_2_)_9_-piperidine		**276** ± 13^*^	**7.97** ± 0.17^*^	9.33

R^3^ = H, unless noted. Data from Kurma et al. [61].

^a^Effect of PAM derivatives on [^35^S]GTPγS binding induced by Cl-IB-MECA [3]. in HEK293 cells (mean ± SEM, n = 3 unless noted otherwise). E_max_ value of Cl-IB-MECA calculated from concentration-response assays expressed as a percentage of vehicle. Values in bold print and demarcated by * indicates P ≤ 0.05 (One-way ANOVA with Dunnett’s T-test for multiple comparisons) with respect to vehicle (i.e., absence of a PAM).

^b^Ratio of the EC_50_ value of Cl-IB-MECA in the presence of 1 μM PAM versus vehicle.

The increasing 2-cycloalkyl ring size, e.g. **23**–**27** and beyond, reached an optimum at 6 – 9 carbons, but outside that range was much weaker [58, 59]. A 2-(cyclohepten-4-yl) group was well tolerated with favorable in vivo pharmacokinetic properties [59]. Bridged substituents, e.g. **30**, performed well. The branched heptyl group in **32** was an improvement over the cycloalkyl groups, but lengthening, shortening, or fluorine substitution of this substituent reduced activity. A 2-phenyl or *N*4-cyclopentyl substitution was not tolerated [60]. The 4-iodo-phenyl substitution of MRS8054 **32** was enhancing, but the 3-iodo substitution prevented PAM activity [60].

This requirement for hydrophobic substitution was puzzling until the putative 1*H*-imidazo[4,5-*c*]quinolin-4-amine binding site was determined (described below in detail) [60–62]. Unexpectedly, the binding site was found to be outside the helical bundle at the intersection of TM7 and helix 8 (H8), with no residues in common with, or even in close proximity to, the orthosteric binding site for adenosine. PAM recognition involves essential interactions with two tyrosine residues in TM7 and H8, largely via π−π stacking, H-bonding contribution from TM1, and hydrophobic interactions with residues in TM1 and 7. This extrahelical site lies closer to the intracellular regions of the receptor that engage directly with G proteins and might represent a conserved signaling nexus for Class A GPCRs. Consistent with this idea, a D_2_ dopamine receptor PAM was subsequently shown to bind at the same site [63].

Recognition that this predicted binding site is lipid-exposed explained the requirement for hydrophobic substitution and subsequently motivated our development of a strategy for secondary anchoring of PAMs within the phospholipid bilayer [60, 61]. Using medicinal chemistry to improve PAM activity, long-chain amino-alkyl congeners of this PAM series were synthesized, including the primary amines **33** and **34**. These analogs were designed based on the prediction that terminal cation groups would form a secondary anchoring interaction at the inner leaflet of the phospholipid bilayer, stabilized by electrostatic interactions with anionic membrane lipids. The resulting “lipid trolling” PAMs exhibited improved pharmacological profiles, including compounds with reduced A_3_AR orthosteric antagonist activity relative to LUF6000. The prototypical A_3_AR PAM LUF6000 increases agonist (adenosine and Cl-IB-MECA) E_max_ but not its potency. In contrast, the long-chain amino-alkyl derivatives enhance both agonist efficacy and potency. For example, MRS8247 **33** increased the E_max_ while reducing the EC_50_ of Cl-IB-MECA at the A_3_AR. We term this strategy *lipid trolling*; that is, “fishing” for stabilizing distal membrane interactions through the attachment of terminal cationic chains to ligands bound at extrahelical sites. This approach is particularly effective for the plasma membrane-embedded A_3_AR, whose cytosolic face is enriched in anionic lipids (e.g., POPS and PIP_2_) relative to the outer leaflet.

In Pradhan et al. [60], the tolerance of substituting the terminal primary amine of compounds **33** and **34** with cyclic amines, such as piperidine and piperazine, was systematically evaluated. These cyclic substituents retained robust PAM activity while exhibiting reduced off-target activity, for example at biogenic amine receptors, compared with the corresponding primary amines. Optimal PAM activity was observed with linker lengths of 6 – 9 methylene units; deviations from this range resulted in diminished activity. Terminal substitution was tolerated only for cationic functionalities, including monoamino, diamino, methylamino, dimethylamino, or guanidino (i.e., **35**) groups. In contrast, neutral or non-cationic substituents, e.g. halo, acetamido, Boc-amino, or even polar hydroxyl groups, were not tolerated. A terminal carboxylate exhibited weak activity only at the highest concentration tested (10 µM). The alkynyl linker present in **33** and **34**, and retained across all subsequent analogues, was essential for activity and could not be replaced by *trans*-alkene or alkane linkers without substantial loss of PAM activity; similarly, introduction of an amide linkage within the chain was detrimental. Relocation of the 8-methylene side chain of **33** from the 4-phenyl to the 3-phenyl position markedly reduced activity. Within the imidazole ring, replacement of *N*3 with thio or oxo functionalities substantially diminished activity, as did replacement of *N*1. Likewise, substitution of the exocyclic 4-NH with methylamino, thio, or oxo resulted in pronounced loss of activity.

In Kurma et al. [61], various reporter groups were conjugated to the terminal position of the amine-functionalized chain. In some cases, *N*-acyl-piperazine was used as the linker to preserve at least one cationic nitrogen in the conjugate. Maintenance of terminal cationic character was essential for retention of PAM activity; consequently, fluorophores containing carboxylic acids were ineffective. Among the conjugates examined, only the rhodamine B derivative MRS8520 **40** retained PAM activity. In addition, a 2-nitroveratryloxycarbonyl (NVOC)-caged derivative **36** was inactive in enhancing A_3_AR agonist (Cl-IB-MECA) responses unless exposed to violet light, which cleaved the photosensitive blocking group and regenerated the required terminal amine.

The PAM activity of the 1*H*-imidazo[4,5-*c*]quinolin-4-amines exhibits marked species differences, with human and canine A_3_ARs showing greater responsiveness than rat and mouse A_3_ARs [61, 64]. For example, MRS8308 **27** increased E_max_ to 348% at the canine A_3_AR at a concentration of 10 µM. Notably, MRS8454 **37** displayed substantial ago-PAM activity, although its E_max_ remained below 100%. Ago-PAM activity was detected using both [^35^S]GTPγS binding and inhibition of forskolin-stimulated cAMP production assays, yielding E_max_ values of 77% of Cl-IB-MECA and 32% relative to the full agonist NECA, respectively.

Unlike the A_1_AR, the biased agonism at the A_3_AR has been reported for orthosteric agonists [65, 66]. LUF6000 displayed biased signaling for adenosine-induced Gα_i3_ and Gα_OA_ isoproteins compared to arrestins [59]. The biased allosteric modulation at various signaling pathways has been carefully examined at the A_3_AR [67, 68]. In cAMP signaling, LUF6000 enhanced the efficacy of all agonists examined, but in the membrane hyperpolarization assay, it only enhanced the efficacy of partial agonists. In calcium mobilization, LUF6000 did not affect the efficacy of the full agonist NECA but was able to switch the nucleoside antagonist 2-chloro-*N*^6^-(3-iodobenzyl)adenosine (MRS542) into a partial agonist. In the translocation of β-arrestin2, the agonist-enhancing effect of LUF6000 was not pronounced. In an assay of ERK1/2 phosphorylation, LUF6000 did not affect Cl-IB-MECA efficacy. The differential effects of LUF6000 on the efficacy and potency of the agonist Cl-IB-MECA in various signaling pathway have been interpreted quantitatively using a mathematical model [68].

## Synthesis routes

**Scheme 1:**
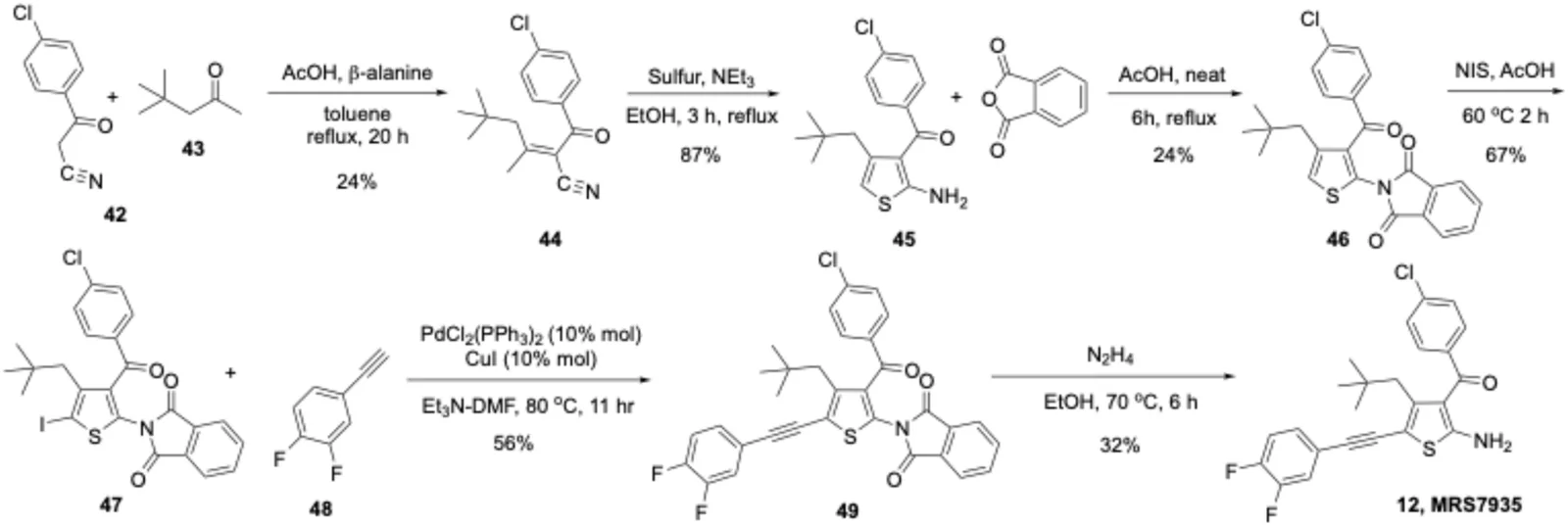
Typical synthesis of A_1_AR PAMs, using **12** as an example

**Scheme 2:**
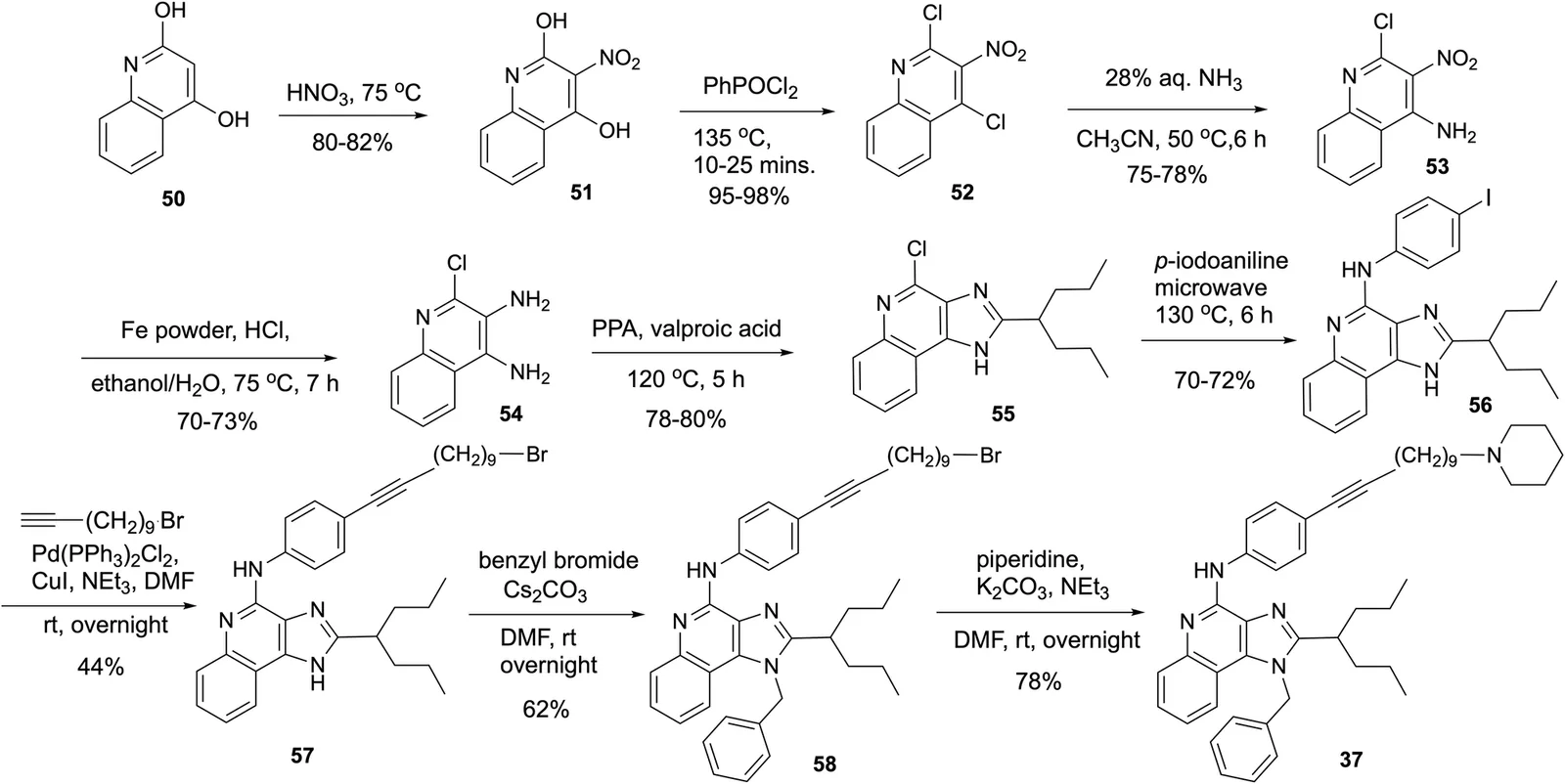
Typical synthesis of A_3_AR PAMs, using ago-PAM **37** as an example [61]

The typical synthetic routes to representative key 2-aminothiophene [20–22] and 1*H*-Imidazo[4,5-c]quinolin-4-amine [9, 58, 60, 61] derivatives are shown in Schemes 1 and 2. The thiophene scaffold of the A_1_AR PAMs was synthesized via a two-step version of the one-pot Gewald reaction—in this case, a Knoevenagel condensation to form an α,β unsaturated ketone **44** from α-cyanoketone **42** and carbonyl precursor **43**, followed by dehydration. This enone **44** was then cyclized in the presence of elemental sulfur via an unelucidated mechanism to yield the thiophene **45**. To prepare the substrate for the acetylene coupling, the 2-amino group was protected (**46**), and a 5-iodo substitution was made via a regioselective electrophilic aromatic substitution using *N*-iodosuccinimide to yield **47**. The iodinated compound was then extended via a Sonogashira cross-coupling reaction of the halide and 4-ethynyl-1,2-difluorobenzene **48**. A deprotection of **49** with hydrazine then afforded the final compound **12**, MRS7935 [21].

A_3_AR ago-PAM **35** was synthesized as outlined in Scheme 2. Initially, intermediates **53–56** were prepared according to reported literature procedures [58, 60]. Quinoline-2,4-diol **50** was first nitrated with nitric acid to afford 3-nitroquinoline-2,4-diol **51**. Compound **51** was then chlorinated with phenylphosphonic dichloride to afford 2,4-dichloro-3-nitroquinoline **52**. Regioselective amination of **52** with 28% aq. ammonia produced 2-chloro-3-nitroquinolin-4-amine **53**. Reduction of the nitro group in **53** using Fe powder and HCl furnished the vicinal diamine 2-chloroquinoline-3,4-diamine **54**. Compound **55** was obtained *via* condensation of the vicinal diamine **54** with valproic acid in the presence of polyphosphoric acid (PPA), resulting in cyclization. A subsequent microwave-assisted reaction between **55** and 4-iodoaniline in ethanol at 130 °C afforded the 1*H*-imidazo[4,5-*c*]quinoline-4-amine derivative **56**. Installation of a long-chain alkyne at the para position of the 4-phenylamino moiety was accomplished using standard Sonogashira coupling conditions (Pd(PPh_3_)_2_Cl_2_, CuI, Et_3_N), yielding compound **57** with 11-bromoundec-1-yne as the coupling partner. *N*1-benzylation of **57** using benzyl bromide and Cs_2_CO_3_ produced the *N*1-benzyl derivative **58**. Finally, treatment of **58** with piperidine in the presence of K_2_CO_3_ and Et_3_N afforded the target terminal piperidine compound **37** [61].

## Structural studies – A_1_ adenosine receptor

**Fig. 4:**
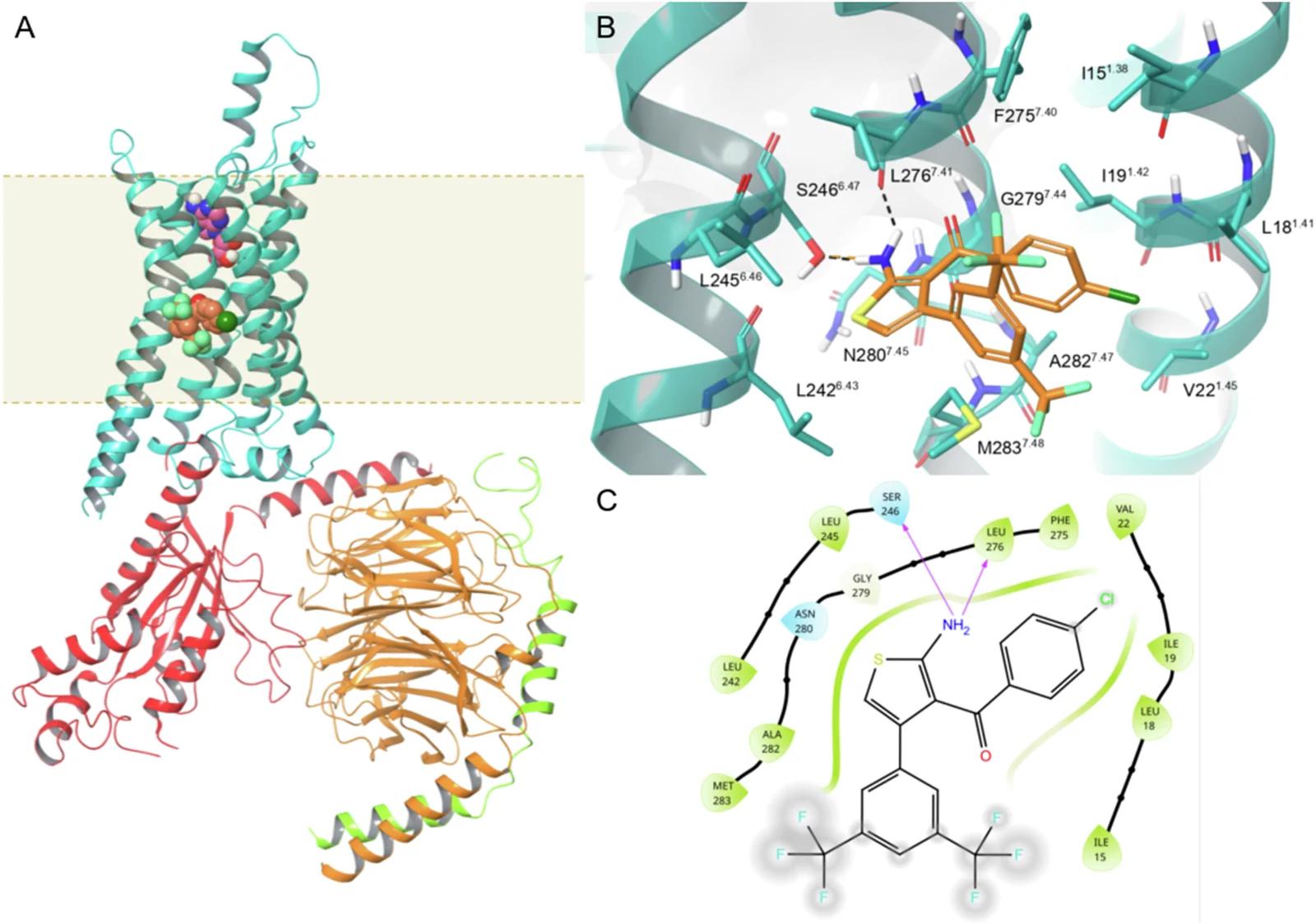
Cryo-EM structure of a PAM **16** bound to the hA_1_AR [13]. **A** hA_1_AR (blue ribbons) in complex with adenosine (pink spheres), MIPS521 (PAM, orange spheres), and the G_i_ protein heterotrimer, with the α (red ribbons), β (orange ribbons), and γ (green ribbons) subunits shown. The approximate position of the plasma membrane is indicated by the brown box. **B** Three-dimensional representation of the MIPS521 binding mode (orange sticks) within the extrahelical allosteric binding site of the hA_1_AR (blue ribbons). Receptor residues within 4 Å of the ligand are shown as blue sticks and labeled using both residue numbering and the Ballesteros-Weinstein GPCR numbering scheme [70]. Hydrogen bonds between the MIPS521 and receptor residues are indicated by black dashed lines. **C** Two-dimensional representation of MIPS521 bound at the extrahelical allosteric site of the hA_1_AR (blue ribbons), highlighting receptor residues within 4 Å of the ligand. Ligand-receptor hydrogen bonds are depicted as purple arrows, hydrophobic interactions are shown as green contour lines, and lipid exposure is indicated by black shadows

Cryo-EM structural determination of the hA_1_AR in complex with the 2-amino-3-benzoylthiophene PAM MIPS521 **16** was reported by Draper-Joyce et al. (PDB ID: 7LD3) [13]. MIPS521 (K_B_ 11 µM, αβ 65) binds an extrahelical, lipid-exposed allosteric site on the A_1_AR (fig. 4A). This allosteric pocket is located at the interface of TM helices 1, 6, and 7 and is largely embedded within the surrounding membrane lipid bilayer (fig. 4B,C). Notably, this site had not previously been targeted by ligands on other GPCRs [69].

MIPS521 is anchored within the allosteric pocket by two key hydrogen bonds formed between its essential amino group and the side chain hydroxyl of S246^6.47^ (using Ballesteros-Weinstein numbering [70]) as well as the backbone carbonyl of L276^7.41^(fig. 4B,C) [13]. Further stabilization is provided by extensive hydrophobic interactions with residues in TM1 (e.g. L18^1.41^, I19^1.42^, V22^1.45^), TM6 (e.g. L242^6.43^, L245^6.46^) and TM7 (e.g. M283^7.48^). The sulfur atom of the thiophene ring is enclosed in a hydrophobic subpocket, forming favorable contacts with L242^6.43^ and L245^6.46^, while the 3,5-bis(trifluoromethyl)phenyl group is exposed to the surrounding membrane lipids (fig. 4B, C). This orientation is consistent with the established role of trifluoromethyl substituents in enhancing binding affinity at hydrophobic sites [71] and improving membrane permeability and lipid partitioning [72]. The ability of MIPS521 to differentially modulate adenosine vs. a novel analgesic A_1_AR agonist was analyzed computationally, pointing to a mechanism involving TM7 dynamics [73].

As previously mentioned, the experimentally defined location of the hA_1_AR allosteric site contrasts sharply with several earlier modeling attempts, which predicted the PAM binding site to reside within the extracellular loop region [74–76]. Guided by this originally proposed and mutagenesis-supported topology [77, 78], nucleoside-PAM conjugates were designed to function as bitopic ligands capable of simultaneously engaging the orthosteric and allosteric sites of the A_1_AR. However, inspection of the cryo-EM structure (fig. 4A) reveals that this binding mode is not feasible, as representative compounds from this series (e.g., **5** and **6**) [44, 45] are not of sufficient length to span the spatial separation between the two sites. Moreover, the extrahelical, membrane-embedded nature of the A_1_AR allosteric pocket would require PAM substituents that are predominantly hydrophobic. This requirement is inconsistent with the SAR observed for this ligand series, further arguing against engagement of the experimentally identified PAM site [79, 80]. Interestingly, iterative structural optimization of compound **6** yielded highly potent and selective agonists for the G_s_-coupled A_2B_AR [81]. This outcome indirectly supports the conclusion that these bitopic ligands do not access the canonical A_1_AR PAM site, as the critical residue S246^6.47^, whose side-chain hydroxyl group forms a key hydrogen bond with the PAM amino group in the A_1_AR, corresponds with C246^6.47^ on the A_2B_AR [82]. The uncertainty regarding the binding site of the PAM moiety in A_1_AR bitopic agonists was ultimately resolved by cryo-EM analysis of **6** bound to the hA_1_AR. As disclosed by A. Christopoulos during the “2024 Goodman and Gillman Award Lecture” [83], this structure revealed that the PAM moiety occupies a distinct extrahelical pocket located between TM6 and TM7, immediately beneath ECL3, rather than the previously characterized TM1/6/7 lipid-facing site [83].

Notably, the cryo-EM structure of the hA_1_AR in complex with PAM **16** enabled the rational discovery of a new class of subtype-selective A_1_AR PAMs. This effort transitioned from the traditional thiophene scaffold to a thiazole-based chemotype through docking-based virtual screening of a 160-million compound library, followed by pharmacological validation and structure-guided hit optimization [36]. Conventional molecular docking calculations suffer from two inherent limitations: inadequate accommodation for receptor conformational flexibility and limited accounting for solvent-mediated interactions [84]. To address the unique challenges posed by the membrane-exposed nature of the A_1_AR allosteric site, the authors implemented an ensemble docking strategy using snapshots of receptor conformations extracted from MD simulations of the MIPS521-A_1_AR complex embedded in an explicit lipid bilayer. Lipid molecules sampled during the simulations were retained as integral components of the binding site during subsequent docking calculations [36]. This protocol explicitly incorporated receptor dynamics and lipid-mediated interactions into the virtual screening pipeline by evaluating ligand binding across multiple independent receptor conformations in the presence of explicit membrane components, thereby substantially improving the biological relevance of the docking predictions.

## Structural studies – A_3_ adenosine receptor

**Fig. 5:**
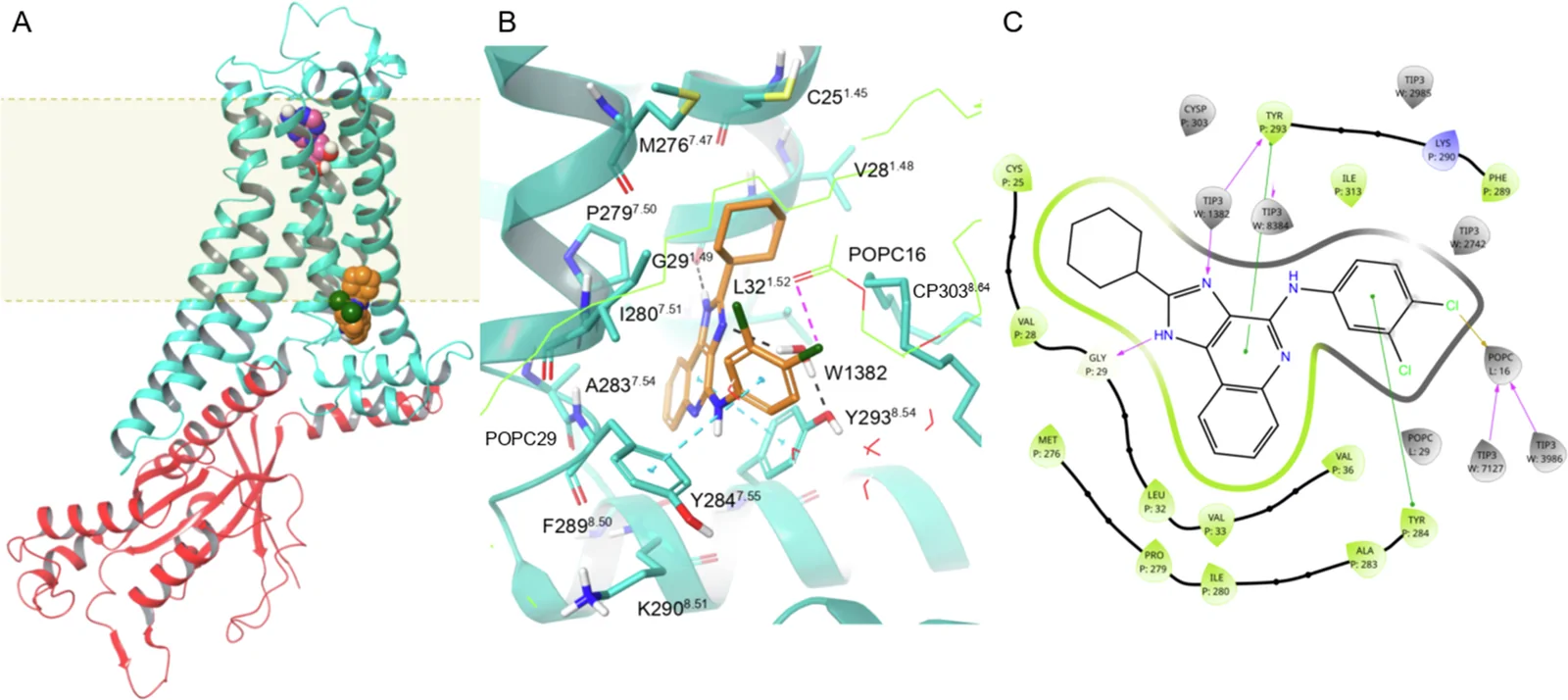
Modeled and MD-validated structure of LUF6000 26 bound to the hA_3_AR [62]. **A** hA_3_AR (blue ribbons) in complex with adenosine (pink spheres), the PAM LUF6000 (orange spheres), and the Gi protein α subunit (red ribbons). The approximate position of the plasma membrane bilayer is indicated by the brown box. The model was generated using an integrated computational pipeline comprising de novo structure prediction, homology modeling, molecular docking, and MD simulations, followed by experimental validation through site-directed mutagenesis and structure-activity relationship expansion [62]. **B** Three-dimensional representation of the modeled binding mode of PAM LUF6000 (orange sticks) within the extrahelical allosteric site of the hA_3_AR (blue ribbons). Receptor residues located within 4 Å of the ligand are shown as blue sticks and labeled using both residue numbering and the Ballesteros-Weinstein (1995) GPCR numbering scheme [70]. Lipid molecules are shown as green sticks and water molecules as red sticks. Hydrogen bonds are depicted as black dashed lines, π-π stacking interactions as blue dashed lines, and halogen bonds as purple dashed lines. **C** Two-dimensional representation of the LUF6000 binding mode in the extrahelical allosteric site of the hA_3_AR (blue ribbons) with residues within 4 Å of the ligand. Hydrogen bond interactions are depicted as purple arrows, π-π stacking interactions as dark green lines, halogen bonds as brown arrows, hydrophobic interactions as light green contour lines, and lipid exposure as black shadows

Despite the recent publication of five cryo-EM structures of the hA_3_AR [14, 85] and five structures of the sheep A_3_AR [86], no PAM-bound A_3_AR structure is available to date. Exploiting known species differences [64], Fisher and colleagues investigated the allosteric activity of the prototypical 1*H*-imidazo[4,5-*c*]quinolin-4-amine PAM LUF6000 (**26**) using a series of human/mouse chimeric A_3_ARs [59]. These studies localized the PAM binding site to the cytoplasmic region of the receptor [59], specifically to the intracellular ends of TM1 and TM7, H8, and the ICL1 [62]. Building on these findings, and in the absence of an experimental A_3_AR structure at the time, the G_i_-bound active-state conformation of the A_3_AR was modeled using a combined de novo prediction and homology modeling strategy, followed by systematic scanning of the receptor surface for druggable cavities [62]. Remarkably, retrospective alignment of this model with the subsequently reported agonist-bound cryo-EM structure (PDB ID: 8X17) [14] revealed a backbone RMSD of 1.4 Å, providing strong validation of the predicted receptor architecture. Modeling calculations predicted a putative allosteric site as an extrahelical, lipid-exposed pocket located at the interface between H8 and the intracellular ends of TM1 and TM7 (fig. 5A) [62]. Notably, this pocket corresponds to a previously described cryptic site that had not been targeted by small molecules [87, 88]. As observed for A_1_ PAMs, this location contradicted earlier modeling studies proposing alternative binding modes for LUF6000 **26** at the A_3_AR [7, 89]. Importantly, this site shares no common residues with the cryo-EM-defined A_1_AR PAM binding site.

To validate the proposed allosteric site and elucidate key determinants of PAM recognition, LUF6000 **26** was docked into the modeled pocket, and the resulting complex was subjected to extensive MD simulations in an explicit membrane environment. These simulations identified a stable bound pose that was fully consistent with prior SAR findings (fig. 5B,C) [62]. Specifically, the ligand’s tricyclic aromatic core engages a face-to-face π-π stacking interaction with Y293^8.54^ and forms a hydrogen bond between its *N*1 atom and the backbone carbonyl of G29^1.49^ (fig. 5). A water-bridged hydrogen bond further links the hydroxyl group of Y293^8.54^ to the ligand *N*3 atom. Further stabilization of the aromatic core is provided by several close-range hydrophobic interactions with L32^1.52^, V33^1.53^, I280^7.51^, A283^7.54^, and F289^8.50^. A second π-π stacking interaction is observed between the *N*4-(3,4-dichlorophenyl) substituent and Y284^7.55^. The *p*-chloro substituent on this phenyl ring forms a halogen bond with the carbonyl of a nearby 1-palmitoyl-2-oleoyl-*sn*-glycero-3-phosphocholine (POPC) molecule, consistent with the emerging role of halogen bonds in membrane-associated ligand interactions and with the unique positioning of the allosteric site at the interface between the inner leaflet of the plasma membrane and the cytosol [90]. The C2-cyclohexyl substituent occupies a shallow, hydrophobic subpocket defined by C25^1.45^, V28^1.48^, G29^1.49^, M276^7.47^, P279^7.50^, and I280^7.51^, in agreement with the ring-size constraints identified in earlier SAR studies [58, 59]. The ligand also establishes favorable hydrophobic contacts with surrounding phospholipids, including the palmitoyl chain of C303^8.64^. The proposed binding mode was validated by site-directed mutagenesis, which highlighted the critical roles of Y284^7.55^ and Y293^8.54^, as well as by SAR expansion studies demonstrating the importance of hydrogen bond-competent nitrogen moieties at the *N*1, *N*3, and *N*4 positions of the ligand scaffold [62].

Interestingly, the experimentally identified hA_3_AR allosteric site is consistent with the G protein-biased signaling profile of this class of PAMs [59]. Extensive ligand interactions with TM7 are predicted to constrain the conformational flexibility of this helix, aligning with multiple independent reports demonstrating that increased TM7 destabilization is typically associated with β-arrestin biased signaling [91, 92]. Notably, this PAM class appears to function as a “molecular glue”, stabilizing H8 in an upward-oriented conformation through interactions with TM1 and TM7. Given that conformational transitions of H8 have been reported to play a central role in class A GPCR activation and G protein coupling [93, 94], stabilization of this helix provides a plausible mechanistic basis for the PAM activity observed for this chemical class.

As described above, two new generations of A_3_AR PAMs were rationally designed by exploiting the localization of the allosteric site at the receptor-membrane lipid. Specifically, alkylamine tails were introduced to enable favorable electrostatic interactions with negatively charged phospholipids that are enriched in the inner leaflet (i.e, lipid trolling) [60, 61, 95]. Notably, atomistic MD simulations revealed that distinct lipid-trolling substituents exhibited differential membrane-ligand interaction profiles, suggesting a tunable influence on binding affinity. Moreover, these simulations demonstrated that incorporating lipid-trolling tails can locally perturb membrane lipid organization near the allosteric site, preferentially enriching phospholipids near TM6, TM7, and H8. This lipid distribution has been reported to prime class A GPCRs for activation [96], offering an additional mechanistic hypothesis for the allosteric activity observed with this PAM class.

Because the 1*H*-imidazo[4,5-*c*]quinolin-4-amine class of PAMs originally derived from AR antagonists, it was not unexpected that the prototypical A_3_AR PAM LUF6000 and many early congeners retained measurable affinity for the orthosteric binding site. This residual antagonist activity tended to attenuate PAM activity. It was associated with the observation that many of the early PAMs in this series enhanced E_max_ without producing a corresponding leftward shift in their EC_50_ values. Consistent with this interpretation, the cyclopropyl analog **23** exhibited exclusively antagonistic activity at the A_3_AR. Indeed, molecular modeling has indicated that many A_3_PAMs, including **23**, can adopt energetically favorable poses within the orthosteric pocket that overlap with canonical AR ligand orientations [58]. These poses preserve key interactions required for both agonist and antagonist binding, including hydrogen bonding with N250^6.55^ and π-π stacking with F168^ECL2^ [97–100]. Notably, the imidazole *N*1 atom was predicted to project toward the hydrophilic ribose binding region of the A_3_AR orthosteric site. On this basis, Kurma et al. [61] explored *N*1 alkylation with bulky hydrophobic groups as a strategy to reduce orthosteric site engagement while preserving affinity for the allosteric site. Molecular modeling predicted *N*1 modification would disrupt orthosteric binding due to unfavorable steric and electrostatic complementarity, while exerting minimal impact on allosteric site interactions [61]. Consistent with these predictions, benzyl substitution at *N*1 in compounds **37**–**40** effectively eliminated detectable antagonist activity and resulted in marked enhancement of both the EC_50_ and E_max_ of Cl-IB-MECA. Furthermore, introduction of a bulky, hydrophobic *p*-iodo substituent on the *N*-benzyl group in **41** produced an additional increase in agonist potency.

## Conclusions

Coincidentally, two distinct series of empirically discovered PAMs for the G_i_ protein-coupled A_1_ and A_3_ARs, identified decades ago, were later found to bind to different extrahelical sites. Contradicting earlier modeling efforts, the lipid-exposed locations were based on structural determination by cryo-EM (A_1_) and by mutagenesis-based molecular modeling (A_3_). The A_3_AR site is close to the cytosolic region that engages the G protein, including H8 and TMs 7 and 1, while the A_1_AR PAM site is close to the orthosteric binding site, but on the opposite side of TMs 5 and 6. In both cases, these sites are largely embedded within the hydrophobic environment of the membrane bilayer, which may explain the inherent hydrophobicity required for ligand activity with each series. Moreover, because the 2-amino-3-benzoylthiophene PAM site on the A_1_AR is spatially and structurally distinct from the binding site of the 1*H*-imidazo[4,5-*c*]quinolin-4-amine PAMs on the A_3_AR, the pronounced subtype selectivity observed for each chemotype is readily explained. For the A_3_AR PAM series, partial engagement of the orthosteric binding site results in residual antagonist activity that would limit allosteric enhancement. Importantly, this liability can be mitigated through strategic scaffold functionalization, specifically by introducing bulky hydrophobic substituents at the *N*1 position to sterically block access to the ribose binding region of the orthosteric pocket. This approach yields a marked improvement in the pharmacological profile of the A_3_AR PAMs. The modeling-based putative location of this A_3_AR allosteric site, in a potential signaling hotspot, is supported by multiple lines of experimental evidence, including SARs, mutagenesis, and its prior designation as a cryptic binding site for Class A GPCRs. The remaining challenge is to advance PAMs from both chemotypes toward translational development. Targeting the spatiotemporal activation of either the A_1_ or A_3_ARs offers distinct therapeutic advantages, including the potential for improved efficacy and safety in indications such as chronic pain.

## Data Availability

No datasets were generated or analysed during the current study.
